# Advances and challenges in ecological connectivity science

**DOI:** 10.1002/ece3.70231

**Published:** 2024-09-01

**Authors:** Amanda R. Liczner, Richard Pither, Joseph R. Bennett, Jeff Bowman, Kimberly R. Hall, Robert J. Fletcher, Adam T. Ford, Julia L. Michalak, Bronwyn Rayfield, Julian Wittische, Jason Pither

**Affiliations:** ^1^ Okanagan Institute for Biodiversity, Resilience and Ecosystem Services University of British Columbia Kelowna British Columbia Canada; ^2^ National Wildlife Research Centre Environment and Climate Change Canada Ottawa Ontario Canada; ^3^ Department of Biology Carleton University Ottawa Ontario Canada; ^4^ Wildlife Research and Monitoring Section Ontario Ministry of Natural Resources and Forestry Peterborough Ontario Canada; ^5^ North America Science The Nature Conservancy Haslett Michigan USA; ^6^ Department of Wildlife Ecology and Conservation University of Florida Gainesville Florida USA; ^7^ Department of Biology, Irving K. Barber Faculty of Science University of British Columbia Kelowna British Columbia Canada; ^8^ School of Environmental and Forest Sciences University of Washington Seattle Washington USA; ^9^ Apex Resource Management Solutions Ottawa Ontario Canada; ^10^ National Museum of Natural History Luxembourg Luxembourg; ^11^ Fondation Faune‐Flore Luxembourg Luxembourg; ^12^ Department of Biological Sciences Complexe Des Sciences Montréal Québec Canada

**Keywords:** biological realism, climate change, connectivity conservation, corridors, directional movement, multi‐species

## Abstract

Maintaining and restoring ecological connectivity will be key in helping to prevent and reverse the loss of biodiversity. Fortunately, a growing body of research conducted over the last few decades has advanced our understanding of connectivity science, which will help inform evidence‐based connectivity conservation actions. Increases in data availability and computing capacity have helped to dramatically increase our ability to model functional connectivity using more sophisticated models. Keeping track of these advances can be difficult, even for connectivity scientists and practitioners. In this article, we highlight some key advances from the past decade and outline many of the remaining challenges. We describe the efforts to increase the biological realism of connectivity models by, for example, isolating movement behaviors, population parameters, directional movements, and the effects of climate change. We also discuss considerations of when to model connectivity for focal or multiple species. Finally, we reflect on how to account for uncertainty and increase the transparency and reproducibility of connectivity research and discuss situations where decisions may require forgoing sophistication for more simple approaches.

## Graphical abstract

A graphical representation of the important connectivity concepts discussed in our review and how they relate to each other. We discuss how connectivity models have increased in biological realism and why that is important, advances in directional and climate connectivity as well as multi‐ and single‐species connectivity models.

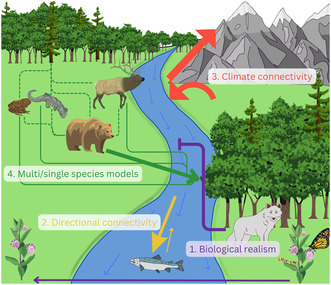

## 1 INTRODUCTION

At the 2022 United Nations (UN) Conference of Parties for the Convention on Biological Diversity (CBD), countries from around the world agreed to a new framework to help reverse the decline of biodiversity. The framework includes four goals and 23 targets, of which five targets either explicitly or implicitly include the need for maintaining or enhancing ecological connectivity. Indeed, the primary long‐term goal (A) is for countries to take action to ensure that the “integrity, connectivity and resilience of all ecosystems are maintained, enhanced, or restored, substantially increasing the area of natural ecosystems by 2050” (CBD, 2023). Countries now face the challenge of understanding how to measure, monitor, model, and restore connectivity in their efforts to meet their commitments.

It has been 40 years since Gray Merriam (1984) first proposed the concept of what was called “landscape connectivity” at the time, defining it as “the degree to which absolute isolation is prevented by landscape elements which allow organisms to move among habitat patches.” Since that time there have been a variety of definitions (e.g., Ament et al., 2014; Taylor et al., 1993; With et al., 1997) including a recent one adopted by Parties to the UN Convention on Migratory Species (CMS 2024) that defines ecological connectivity as being “the unimpeded movement of species, connection of habitats without hindrance and the flow of natural processes that sustain life on Earth.”

No matter the definition, connectivity is understood to be essential for a variety of ecological processes, including daily foraging, dispersal, gene flow, migration, metapopulation dynamics, species' abilities to shift ranges and adapt to the effects of climate change, and ultimately population persistence (Fletcher et al., 2016; Hilty et al., 2020). Connectivity is affected by the structure of the landscape (i.e., the sizes, shapes, compositions, elevational gradients, and arrangements of landscape components) and the abilities of organisms to move through the various components of the landscape. These are known as structural and functional connectivity, respectively (Hilty et al., 2020; Merriam, 1984); and although the former is relatively easy to measure, the latter is by definition species‐specific and therefore more challenging to model.

Our understanding of ways to model functional connectivity has increased dramatically over the last decade thanks to an increase in data availability and methodological advances, allowing for a rapid increase in the amount of relevant research (Correa Ayram et al., 2016; Hall et al., 2021; Wade et al., 2015). A simple Google Scholar search for articles with “landscape connectivity” returned over 330,000 results since 2014. The diversity of methodologies and tools leveraged by connectivity research has likewise expanded rapidly in recent years. This expansion reflects a broadening of the focal scales and breadth of movement‐related processes being addressed, as well as advances in tools and computational power. Keeping apprised of these developments—even in one's area of expertise—is becoming increasingly challenging.

We address this challenge by focusing our review on two areas of rapid advancement (1) incorporation of more realism into connectivity models and (2) extensions from single species to multiple species. This review is particularly well‐suited for researchers and practitioners to get introduced to connectivity science, as well as for managers and funders to decide where to prioritize conservation funding and efforts. The issues explored in this synthesis reflect the expertise of presenters who participated in a symposium together in 2020. The four sections of this review explore approaches aimed at improving the biological realism of connectivity models, including incorporating population processes, and individual/species‐specific aspects of movement (Section 2), movement directionality (Section 3), the impact of climate change on connectivity (Section 4), and examining the practicalities of single‐ versus multi‐species analyses for connectivity assessments (Section 5). We conclude by recommending areas where future research should focus on advancing connectivity science. We assume the reader has a baseline understanding of connectivity science in this synthesis; useful primers that introduce essential terms and topics in connectivity science include Rudnick et al. (2012) and Hilty et al. (2020).

## 2 INCREASING THE BIOLOGICAL REALISM OF CONNECTIVITY ANALYSES FOR SPECIES AND/OR INDIVIDUALS

Connectivity models span a gradient of biological realism (Calabrese & Fagan, 2004). Simplified approaches to connectivity models such as identifying the most efficient movement route (i.e., least cost path) between focal patches, or the range and relative likelihood of potential pathways in response to a modeled landscape (i.e., Circuitscape). These approaches facilitate the modeling of movement processes and can be used alone or in combination to consider many types of conservation and management questions. However, their departure from biological realism (Diniz et al., 2020; Sawyer et al., 2011) introduces uncertainty in the effectiveness and relevance of connectivity models to conservation (Beier et al., 2009; Fahrig et al., 2021; Hodgson et al., 2009), especially for species where simplifying assumptions (e.g., assuming any forested area will facilitate movement for all species) are unlikely to be appropriate. Incorporating more biological realism into connectivity models has been championed to address simplifying assumptions (LaPoint et al., 2013), potentially reducing uncertainty between model outputs and true movement paths of focal organisms and increasing the predictive accuracy of connectivity actions (Poli et al., 2020). This may ultimately lead to more effective conservation decisions (Aben et al., 2016; Robertson et al., 2018; Vasudev et al., 2015). However, there may be cost trade‐offs in adding such realism, both in terms of financial cost of gathering more information and in terms of delays to management action (McDonald‐Madden et al., 2010).

There have been important advances in increasing the realism of connectivity models in recent years. We organized these advances into four key areas: (1) isolating relevant movement behaviors or processes that drive connectivity, (2) acknowledging population and community‐level parameters that may alter model outcomes, (3) capturing the complexity of landscape attributes relevant to connectivity in both space and time, and (4) improving the flexibility of algorithms used to model connectivity.

First, methods that can isolate movement behaviors and processes important for connectivity are a key advancement for increasing biological realism. Such methods include simulated individual movement paths (Whittington et al., 2022) and hidden Markov models (see McClintock et al., 2020; Glennie et al., 2023 for overviews of these models) to identify “exploratory” or “dispersive” components of GPS tracks (Harju et al., 2013). Exploratory components tend to reflect directed movement (i.e., large step lengths and small turning angles) that are thought to be relevant to organisms dispersing across landscapes. The behavior and movement of organisms may also be influenced by human behaviors or “anthropogenic resistance” (Ghoddousi et al., 2021; Williamson et al., 2023) which in turn would impact connectivity and should be considered. By identifying realistic intrinsic and human modified behavioral states, resistance (or cost/friction) maps can be refined to predict resistances on those movement components that we expect are driving actual connectivity across real landscapes.

Second, incorporating known species distributions or population sizes into connectivity models, which has been termed “demographic weighting” (Drake et al., 2021), provides a way for connectivity assessments to better account for movement potential and dispersal across landscapes. For instance, improving our understanding of how propagules move through the landscape can in turn inform models and help identify important connectivity areas. Similarly, species distribution models (SDMs) can be used with movement models to incorporate both high‐quality habitat and important movement corridors into conservation planning (Almpanidou et al., 2014; Puddu & Maiorano, 2016). For example, Almpanidou et al. (2014) found that areas of high‐quality habitat identified using SDM were not the same areas used for brown bear movement. Puddu and Maiorano (2016) found that when both habitat suitability and structural connectivity were considered, areas of suitability for mouflon were reduced. Related population and community dynamics, such as social behavior within and among species (Zeigler et al., 2011) or variation in movement tendency among individuals (Sullivan et al., 2021), have also been incorporated to refine expectations for when or where habitat connectivity supports key processes or interactions. Using hierarchical models that incorporate post‐dispersal reproduction emphasizes the importance of recruitment and gene flow as key outcomes of movement, a modeling advance that has been termed “effective connectivity” defined as “connectivity that is followed by the successful reproduction of immigrants” (Robertson et al., 2018; Van Moorter et al., 2021).

Third, connectivity science is getting better at capturing real landscape complexity. There is an increasing focus on landscape dynamics and their relevance to connectivity and conservation (Bishop‐Taylor et al., 2018; Zeller et al., 2020). Improved computer processing capability has enabled greater use of fine‐grained spatial data, data with a greater thematic resolution, and consideration of complex features (e.g., slope, microclimate, Nowakowski et al., 2015).

**FIGURE 1:**
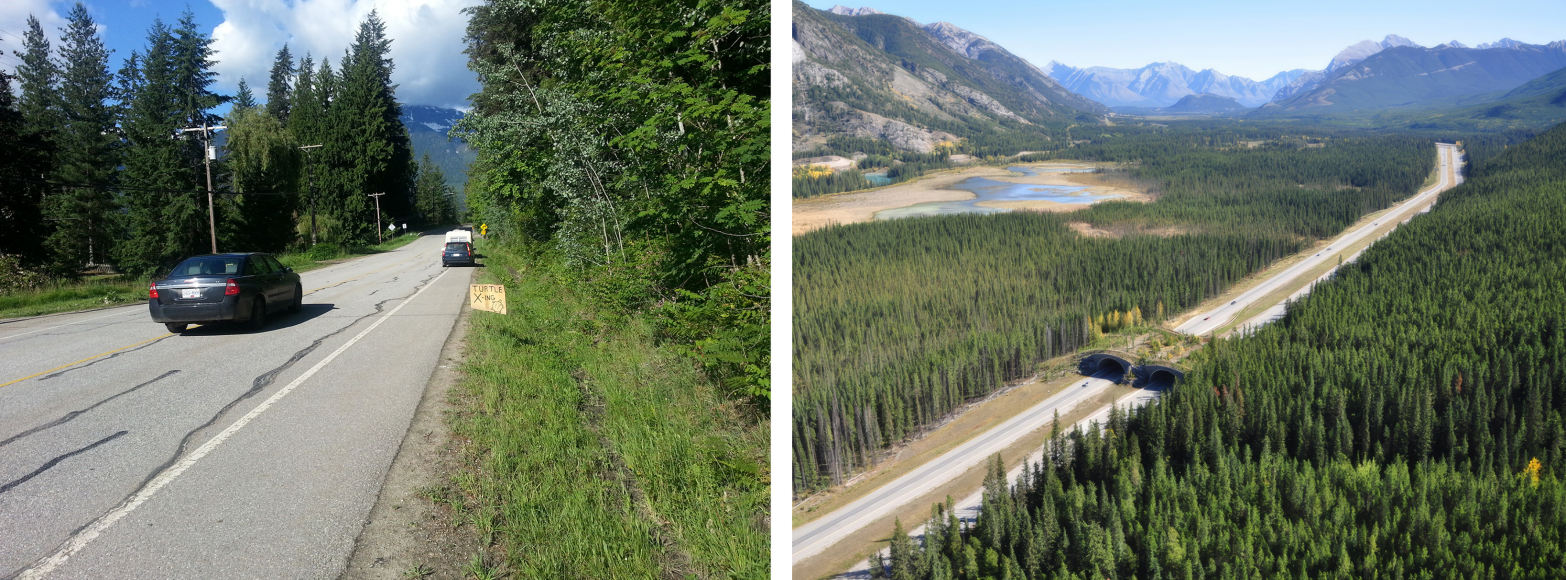
Examples of real‐world connectivity conservation actions at different levels of effort. The left image shows a community‐made road sign informing drivers of a prominent turtle crossing area. The right shows a wildlife crossing over a freeway. Photo credit: Adam T. Ford.

Finally, connectivity algorithms have improved so that we can better represent biological realities. Temporally explicit connectivity metrics are more common (Martensen et al., 2017), as are circuit theory extensions using Markov chains (Fletcher et al., 2019) that enable the variability of real biological traits to be modeled through connectivity mapping (e.g., species distribution, mortality, movement directionality). The spatial absorbing Markov chain (SAMC, see Marx et al., 2020 for an overview of this method) was designed, in part, to depict movement failures, such as road mortality or human–wildlife conflict (although we note that this framework can be applied more broadly to situations that may or may not include movement failure). In this way, it isolates the effect of landscapes on movement behavior (via resistance) and mortality (via “absorption”), recognizing that movements can terminate due to settlement or residency occurring after dispersal. Mortality has a variety of sources, such as known risk of vehicle collision locations, natural events based on known survival estimates, or relationships with key landscape features, such as the human footprint (Hill et al., 2020; Marx et al., 2020). Understanding these patterns can improve our understanding of land change impacts on population viability and help identify conservation strategies (Figure 1, Yamaura et al., 2022). Individual‐based models are also a means to consider biologically realistic variation in behavior among individuals, sexes, classes, etc. (Aben et al., 2014; Hauenstein et al., 2019).

Of course, challenges to representing all biological realities (seasonal, behaviors, individual, population, etc.) related to movement remain. This movement information could inform connectivity models, yet for most organisms, this information is unknown. Connectivity assessments are urgently required, often faster than new information representing biological realities can be acquired. This leads to two questions worthy of future investigation: how much realism is necessary for effective connectivity assessments? How can we generalize connectivity models across systems and taxa while increasing realism? Evaluating models with observed movements can help answer this first question. For example, Hudgens et al. (2012) found that the value of increased realism and complexity in models was dependent on how the model was used and evaluated: based on model validation, complex models best predicted observed dispersal, and yet simple models based solely on geographic distance explained almost the same amount of variation in observed dispersal. Poli et al. (2020) found that, in an experimental system, incorporating knowledge of habitat quality and expected dispersal distances was necessary for accurate connectivity predictions, but matrix variability had inconsistent effects on predictions.

For the second question, it may be possible, in some cases, to compare habitat suitability to matrix conditions to infer movement behavior of organisms. Eycott et al. (2012) found that movement tended to be greater through matrix conditions that were more similar to a given species' habitat. However, this relationship is complex, as different movement components (e.g., movement probability into a matrix type versus movement speed) may vary with the matrix (Kuefler et al., 2010; Stevens et al., 2004; Stevens, Leboulengé, et al., 2006; Stevens, Verkenne, et al., 2006). Morphological traits could also help generalize and improve realism of connectivity models. Hartfelder et al. (2020) used allometric scaling of dispersal based on morphology to predict connectivity across an entire bird community and found that model predictions explained substantial variation across a protected area network in southern Africa. This kind of trait‐based approach could be useful for extending realistic single‐species connectivity models across communities, but more work is needed to identify traits relevant to connectivity models. Despite these advances, it remains unclear which general principles capture biological realities to improve connectivity science.

Incorporating biological realities may advance connectivity science in two fundamental ways. First, it may improve and expand the scope of connectivity conservation efforts. Several strategies have been proposed that tailor conservation actions based on expectations for how movement is limited across landscapes (Vasudev et al., 2015). For example, connectivity could be limited due to habitat isolation or due to conflict‐induced mortality, each of which requires different mitigation strategies (Vasudev et al., 2023). Second, it may advance how connectivity is monitored and evaluated from the standpoint of effective connectivity, a process that remains unclear when structural connectivity measures are used. Connectivity assessments should strive to ensure models accurately capture real movement behavior and appropriately articulate the limitations and potential implications of modeling choices when constrained by data or knowledge. All connectivity models should be validated but especially predictive/simulated models and models based on limited biologically realistic data (Foltete et al., 2020; Riordan‐Short et al., 2023). Ultimately, the degree to which models are crafted to reflect biological realities should be driven by project objectives and necessary trade‐offs between obtaining high realism vs. resources available to model with a high degree of realism (Diniz et al., 2020).

## 3 DIRECTIONAL, OMNIDIRECTIONAL, AND ANISOTROPIC APPROACHES TO ASSESSING CONNECTIVITY

Advances in incorporating biological realism in connectivity models include approaches for modeling directionality and anisotropy (i.e., the dependence on direction) of movement. Here, we treat the related concepts of directionality and anisotropy separately to distinguish conceptual and technological advances in modeling real movement patterns.

Traditionally, researchers have often focused on movement between predefined source and destination locations, what we refer to as “directional movement”. The least‐cost and randomized least cost path models are examples of directional methods that estimate the optimal route between a source and a destination across a resistance surface (Adriaensen et al., 2003; Kivimäki et al., 2014; Saerens et al., 2009). Similarly, pairwise applications of circuit theory consider movement potential between source and destination nodes, providing both a measure of effective resistance that is a key measure in landscape genetics applications, and a map that represents the cumulative “current densities” as movement proceeds following random walk assumptions across multiple paths on the resistance surface (McRae & Beier, 2007). With either of these methods, multiple nodes can be connected to produce a connectivity network (Etherington, 2016) and be used, for example, to predict connectivity networks among parks and protected areas (e.g., Barnett & Belote, 2021). However, when using predefined nodes in a directional framework, the true source and/or destination may be unknown, overestimating movement probability near nodes (Koen et al., 2014). Consequently, there are some scenarios where a directional approach to connectivity modeling may be problematic.

In the past decade, the variety of connectivity methods has increased for modell individual movements in all directions as opposed to simply node‐to‐node (Koen et al., 2014; McRae et al., 2016; Pelletier et al., 2014) Four main methods have been used for such omnidirectional connectivity: (1) point‐based Circuitscape (where nodes are points on the study area periphery; Koen et al., 2014) (2) factorial least‐cost paths which integrate multiple paths to create a network of connectivity (Cushman et al., 2009), (3) wall‐to‐wall circuit‐theory approaches, where nodes are arranged along edges of tile(s) encompassing the study area (Pelletier et al., 2014), and (4) moving window analysis where every pixel (or a regular sample of pixels) acts as a source and target node within multiple assessments that are summed together (Omniscape, McRae et al., 2016; Landau et al., 2021). These methods have different computer processing requirements but when parameterized similarly, produce similar results (Phillips et al., 2021). Omnidirectional models are also relevant both when used and usable core habitats, or discrete populations, are not easily identifiable, or when landscape connectivity is to be modeled for complex groups such as for multiple species (see Section 4; Pelletier et al., 2014; Koen et al., 2014; Marrotte et al., 2017). This is because maps produced through omnidirectional methods are not constrained by source and destination node placement; Omniscape has the additional flexibility of allowing different source strengths to be associated with each node. Additionally, omnidirectional approaches may be preferred when a species is distributed across a landscape without geographically discrete populations, where specific source and destination nodes are not considered. Species distribution or habitat suitability models could be used to identify source and destination nodes, but more research is needed to accurately link these distribution or habitat suitability models with connectivity models to ensure they represent actual movement routes.

Seascape genetics (White et al., 2010), river systems (Cushman et al., 2014), wind‐aided colonization (Muñoz et al. 2004), and disease ecology simulation studies (Landguth et al., 2017) are pioneering the integration of directionality into connectivity models. Wind has been shown to drive broad‐scale patterns of genetic variation in forests across the globe (Kling & Ackerly, 2021). Similarly, anisotropic measures of oceanographic connectivity have improved our understanding of dispersal in marine organisms (Jahnke et al., 2018; Munguia‐Vega et al., 2018). Changes to wind and current patterns will substantially affect the population dynamics of many species (Henry et al., 2015; Kling & Ackerly, 2020; Storch & Pringle, 2018), particularly as climate change alters oceanic currents (Hu et al., 2020) and wind speeds (Wu et al., 2018; Young & Ribal, 2019). Tools allowing for the creation of explicitly asymmetric estimates of structural connectivity, gene flow, or dispersal have advanced our ability to model anisotropic connectivity (e.g., Fletcher Jr. et al., 2022; Lim et al., 2021; Mims et al., 2019). This will permit studies to overcome ecological biases in models that overestimate (for upstream sites), or underestimate connectivity compared to real‐world movement (Beger et al., 2010; Paz‐Vinas et al., 2013; Treml et al., 2008; Trotter et al., 2019). However, these methods tend to be quite complex, requiring extensive and often interdisciplinary expertise (e.g., computational fluid dynamics) to parameterize anisotropic landscape connectivity.

There are technological gaps in modeling anisotropy that continue to hamper assessing landscape connectivity and prevent more realistic estimates of movement. In circuit theory, the transitions between adjacent pixels or nodes are represented as resistors that do not allow directionality. However, directional forces (e.g., wind and water currents) naturally induce dispersal anisotropy for many species. Directional forces occur simultaneously with landscape influences on movement that resist or promote movement, necessitating the ability to assign multiple values to a landscape cell, depending on the direction (i.e., lower resistance values when moving with the water current vs. against). Anthropogenic infrastructure such as dams (Paz‐Vinas et al., 2013) and transport networks (Bullock et al., 2018) may be enhancing or creating dispersal anisotropy. Nevertheless, with growing interest in and ability to model anisotropy in movement, the gap between conventional connectivity models and those accommodating more biological realism will continue to close.

Changes in modeling directional and anisotropic movement continue on many fronts, including the advance of statistical frameworks that relax previously restrictive model assumptions (Fletcher et al., 2019). Some graph‐theoretic models have also been extended to allow for anisotropy (Huang et al., 2020; Saura, 2010). Additionally, spatial absorbing Markov chains (see Section 1 and Fletcher et al., 2019) extend circuit theory and allow for anisotropic measures of landscape resistance to be readily used in connectivity models. These methods enable innovation and will undoubtedly help achieve better biological realism in connectivity models.

## 4 MODELING CONNECTIVITY IN THE FACE OF CLIMATE CHANGE

Predicting and accounting for changing landscapes is an important step toward improving the realism of connectivity models. Climate change, in particular, is increasing the need for connected landscapes that will allow individuals and species access to locations that retain or develop suitable climatic conditions (Heller & Zavaleta, 2009). Climate change fundamentally alters movement dynamics by enforcing directionality corresponding to shifting landscape conditions (Krosby et al., 2010). Given the unique dynamics of climate‐driven movement, traditional connectivity models may not identify routes appropriate for climate‐driven range shifts (Littlefield et al., 2019). Such climate‐driven movement considerations include: (1) the directional influence of climatic gradients, (2) uncertainties associated with the location of future suitable climatic (and habitat) conditions, and (3) the suitability of climatic conditions along the movement route. Identifying models to address these additional considerations has been a focus of the rapidly evolving field of climate connectivity science. In recent years, ecologists have developed a wide array of modeling approaches to address these challenges that have been reviewed elsewhere (Keeley et al., 2018; Littlefield et al., 2019). Here, we briefly summarize the main types of climate connectivity modeling approaches and then review the more recent articles that have compared approaches in real landscapes, highlighting climate connectivity.

Littlefield et al. (2019) identified four conceptual approaches to climate connectivity modeling. The first relies on principles of climate gradients and shifts and assumes that most species' range shifts will follow climatic gradients poleward and upward in elevation (Anderson et al., 2016, 2023) or prioritize pathways connecting warmer to slightly cooler locations (Nuñez et al., 2013; Krosby et al., 2014; McGuire et al., 2016, p. 201; Gray et al., 2020). The second approach finds pathways based on overlap or routes that connect predicted current‐to‐future range locations for individual species (e.g., McKelvey et al., 2011; Phillips et al., 2008; Rose & Burton, 2009) or multi‐species assemblages (Choe et al., 2021). A third approach identifies climate trajectories by tracking climatic benchmarks as they shift across the landscape. Similar climatic conditions can be connected in incremental chains, identifying connected routes through time (e.g., Burrows et al., 2014) or between disjunct current and future analogs (Carroll et al., 2018; Littlefield et al., 2017). Finally, Beier and Brost (2010) proposed identifying corridors with highly diverse combinations of topography, soils, and geology based on the assumption that such areas will support a diversity of species through time, even if the species using the corridor in the future differ from those present today.

More recently, analysts have shifted from developing new approaches for modeling climate connectivity to comparing results among approaches that do and do not incorporate climate. Several important insights have been gained from these efforts. First, although there is overlap, climate connectivity modeling generally identifies different movement routes compared to traditional connectivity models. For example, Littlefield et al. (2017) found that the model for the western United States (USA) that linked current and future climate analogs prioritized locations with steep climatic gradients and de‐emphasized connectivity in topographically flat regions such as the Columbia Plateau in Washington State (USA). Similarly, Schloss et al. (2021) found that climate connectivity models identified distinct locations in less developed landscapes that were not differentiated by the non‐climate model. However, they found substantial overlap between climate and non‐climate connectivity in highly developed landscapes where options for movement were more constrained.

Several studies have also compared climate connectivity modeling approaches to one another and found very low levels of overlap among locations prioritized by each model. Choe et al. (2021) compared four climate connectivity models in California. Of the four models used, two used different methods to connect current and projected future species locations: one modeled corridors with diverse geophysical characteristics between protected areas, and the second used Omniscape to connect current and future climate analogs while also prioritizing locations with high topographic complexity. The latter study found that nearly 75% of the landscape was prioritized by one or more methods, but only 9.5% of the landscape was prioritized by three or more models. Notably, areas with overlap were largely riparian habitats. However, the authors note caution is needed in interpreting these results as these connectivity models were developed independently and not with comparison in mind. Correspondingly, the models differ in resolution, extent, and methodology. In addition, each model included multiple types of weights and assumptions making it difficult to identify what ultimately drives the differences in spatial prioritization. Similarly, Gray et al. (2020) identified riparian and terrestrial links between protected areas in Northern California. They quantified the climate benefit of each linkage by calculating the temperature differential between each pair of protected areas, the larger the differential, the greater the climate benefit of that linkage. Interestingly, there was very little spatial overlap between the linkages that provided the most cooling benefit based on summer temperature and those that were most beneficial based on winter temperatures. This disparity suggests that climate variables used to define a climate gradient should be carefully selected as this choice could substantially alter prioritization outcomes.

Overall, much remains to be learned about climate connectivity modeling, but several trends are emerging. At large scales, climate connectivity models identify different movement routes compared to non‐climate connectivity models. However, these differences are less pronounced in highly developed regions where connectivity is constrained by the human footprint (Schloss et al., 2021). In addition, climate connectivity modeling is likely to be less informative in flat areas compared to connectivity modeling without considering climate as these regions may lack climate gradients to influence directionality. Topography has such a strong role that areas with steep climatic gradients, including many riparian corridors, are often predicted by multiple models as priorities for climate connectivity. Areas for future research include more deliberate comparisons among climate connectivity approaches, especially to identify general principles in how landscape characteristics influence or relate to climate connectivity. In addition, studies are needed to determine how selected climate variables impact priorities based on analog and gradient‐based approaches and which variables may be most influential for which species. Finally, the field would benefit from a detailed examination of how individual species' climatic sensitivities, dispersal mechanisms and behaviors, and habitat requirements influence movement in response to climate change.

## 5 FOCAL SPECIES AND MULTI‐SPECIES APPROACHES TO MODELING LARGE‐SCALE CONNECTIVITY: THE BENEFITS OF EACH APPROACH

Movement complexity is a significant issue for modeling connectivity for single species, which is amplified for multiple species. The application of single‐species connectivity models to conservation planning is often required for species‐level management or protection, such as critical habitat mapping for species at risk (e.g., Canada lynx [*Lynx canadensis*]; Squires et al., 2013). Single‐species models may also be used as a surrogate or umbrella, under the assumption that a species may protect a regional community (Beier et al., 2006). This assumption requires validation as it may not protect all species in the community (Brennan et al., 2020; Cushman & Landguth, 2012; Meurant et al., 2018). Current research priorities for single‐species connectivity studies include assessing relationships between connectivity and population persistence (Reichert et al., 2021), how forecasted changes to connectivity arising from environmental or anthropogenic change could influence persistence (Zeller et al., 2020), the development of methods to apply these connectivity models to conservation and land use planning, evidence‐based guidance on corridor width, and how to manage corridors to ensure permeability (Daigle et al., 2020; Keeley et al., 2019).

Conservation planning is increasingly moving away from single‐species conservation to targeting biodiversity, requiring consideration of connectivity for multiple species (Koen et al., 2014). Multi‐species connectivity analyses approach this by directly (species measurements) or indirectly (surrogates) assessing landscape connectivity for multiple species. A recent review by Wood et al. (2022) identified that the common goal of these analyses is to support the long‐term persistence of a set of species, requiring the collapse of information on multiple species' habitats or movements. Upstream methods collapse information before calculating connectivity, resulting in a single connectivity map that represents the needs of multiple species (e.g., multi‐species resistance surface [Koen et al., 2014]; naturalness or human footprint approaches [Theobald, 2013, Anderson et al., 2023]; species‐agnostic approaches [Marrec et al., 2020]). Downstream methods map connectivity for individual focal species and then collapse them to arrive at a composite multispecies map (e.g., a stack of individual maps [Cushman & Landguth, 2012]), a union of all focal movement pathways (Liu et al., 2018), or common potential dispersal areas (Albert et al., 2017).

Upstream methods have the advantages of fewer runs and the ability to use readily available data while downstream methods may, in theory, better represent dispersal and connectivity for species with unique needs as they retain more species‐specific information. As novel multi‐species connectivity methods are developed, they should be evaluated not only on their position along this continuum of data and computing resource requirements but also on how well they are validated with empirical observations. For example, community‐level networks proposed by Hartfelder et al. (2020) develop spatial networks from species' allometric scaling and explain substantial variation in bird species richness in protected areas. Current research priorities for multispecies connectivity analyses include: evaluating the model's ability to capture a range of species' connectivity needs and ecological processes, comparing multispecies methods for efficiency (cost, effort) and effectiveness (biological realism), use of multispecies empirical data for model validation, and forecasting multispecies connectivity with environmental and anthropogenic change.

The preferred method to model connectivity (either single‐ or multi‐species) will depend on the objectives, data availability, and time constraints. In some cases, legislative requirements may dictate the approach, including where land use planning requires multi‐species connectivity (Koen et al., 2014). In contrast, species‐at‐risk legislation often requires single‐species connectivity maps (e.g., Gubbi et al., 2016; Solmundson et al., 2020). Implementing strategies based on connectivity modeling requires choices of which species or processes to emphasize in conservation or management plans, as taking action to benefit one focal species or group may reduce connectivity for others. Being able to compare multiple models helps us understand these trade‐offs, and to identify situations where investments to increase realism are most important. Regardless, land managers, stakeholders, and rights‐holders need to have access to simple, effective, and practical guidance on how to enhance connectivity in their areas of interest (Theobald et al., 2000).

Researchers have been able to validate both single‐ and multi‐species connectivity models, underscoring that either may be appropriate under the correct circumstances. Single‐species model validation has benefitted from a longer research history of these methods. For example, Walpole et al. (2012) found that snow tracks of Canada lynx were more likely to occur in areas predicted by a connectivity model developed with an independent dataset of lynx occurrences. Relatively few studies have attempted to validate multi‐species connectivity models with some notable exceptions (e.g., Brennan et al., 2020; Koen et al., 2014; Marrotte et al., 2017; Pither et al., 2023). These studies have used either roadkill, telemetry, or genetic data to assess whether connectivity estimates predict movement. Several studies have also compared single‐ and multi‐species models within the same study system, often in the context of testing whether a single‐species model can be used as a surrogate or umbrella. The consensus has been that surrogate models may represent a subset of species within a community (Beier & Brost, 2010) but are inadequate at modeling the wider community or making predictions across scales (Brennan et al., 2020; Cushman & Landguth, 2012; Meurant et al., 2018). As ecological similarity (i.e. habitat requirements, movement ability) increases among modeled organisms, model fit might be expected to improve (Brodie et al., 2015).

## 6 DISCUSSION

**FIGURE 2:**
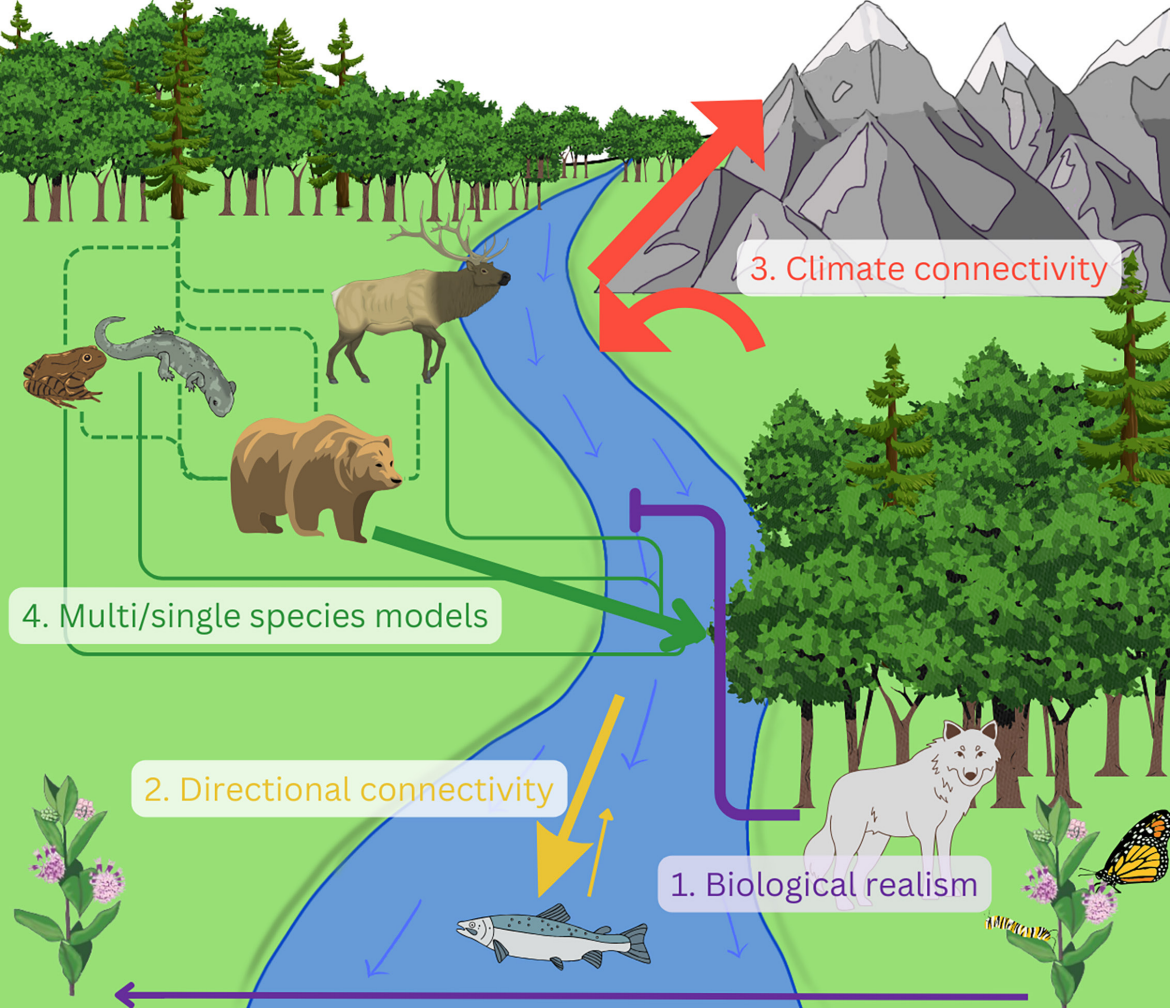
A representation of the broad categories of connectivity described in the manuscript. (1) Biological realism attempts to capture biological realities of connectivity models by considering demographic factors including causes of mortality preventing movement (i.e., predation), and species interactions (i.e., food sources, obligate interactions) influencing movement capacity. (2) Directional connectivity acknowledges that movements may not occur equally in all directions as directional forces (such as water currents) may cause movements to preferentially occur in one direction. (3) Climate connectivity modeling aims to determine where organisms will need to disperse to follow changing climatic conditions such as by shifting elevation or latitude or moving to areas of climate refuge like riparian areas. (4) Multi‐ versus single‐species connectivity modeling considerations. Single‐species modeling may use surrogate or umbrella species in an attempt to capture the movement behavior of many species (dotted lines) or multi‐species modeling considers the movement of multiple species including their different dispersal and habitat needs.

Connectivity science has come a long way since “landscape connectivity” was first coined 40 years ago (Merriam, 1984). Although research in the early years tended to focus on structural connectivity, developments over the past decade, particularly advances in computing hardware and software, have pushed the boundaries of what is technically possible in functional connectivity modeling. The growth of Movebank.org (and similar data platforms) is especially exciting as it has raised awareness of the power and benefits of data sharing and improves the potential to use data for parameterizing models, constructing movement resistance layers, and validating results of connectivity analyses (Kays et al., 2022). In this article, we describe how connectivity models have become more complex and biologically realistic as they attempt to reflect movement direction (omnidirectional and ansiotropic movement; Geoffroy et al., 2019; Phillips et al., 2021; Whittington et al., 2022), isolate different types of movement steps (e.g., Van Moorter et al., 2021), separate mortality from movement (Fletcher et al., 2019), and account for dynamic landscapes, including changes due to the effects of climate change (Arévalo et al., 2020; Carroll et al., 2018). We also describe situations where modeling for either focal or multiple species may be advantageous (Figure 2).

It is important to keep in mind that while improving technical aspects of connectivity modeling is important, it is not the same as improving the accuracy of model predictions, and key gaps in connectivity science remain (Box 1). Most importantly (1) there is still a lack of movement data for many species (Supp et al., 2021), making it difficult to model connectivity (and validate these models) for such species with high confidence. Indeed, many resistance surfaces used in connectivity analyses are constructed by inverting and transforming habitat use data, rather than using movement data to parameterize the surface (Diniz et al., 2020; Keeley et al., 2016; Scharf et al., 2018; Zeller et al., 2012). (2) Sensitivity analyses to investigate the impacts of resistance surface parameterization are needed (Koen et al., 2012; Rayfield et al., 2010). (3) Even for species with movement data, there may be little or no data relating connectivity to population or community dynamics, including population persistence, information that is critical for understanding what “well‐connected” means and accurately prioritizing conservation actions (Drake et al., 2021; Fahrig et al., 2021; Yamaura et al., 2022). Such data, as well as occurrence, road‐crossing, and wildlife‐vehicle collision data are also valuable for validating connectivity analyses (Laliberté & St‐Laurent, 2020; Poli et al., 2020). (4) In addition, basic land cover data used in connectivity analyses is often dated and missing important features such as resource access roads, fences, and road crossing structures (Figure 1, Poley et al., 2022). (5) Similarly, projections of the inevitable expansion of the human footprint and changes to land cover due to climate change and development are important to consider, but usually not available (Littlefield et al., 2019; Zeller et al., 2020).

BOX 1 **Outstanding questions (Q) and needs (N) in connectivity research**

Q1. How much realism is necessary for effective connectivity assessments?

Q2. What components of realism are most important to capture?

Q3. How can we generalize connectivity models across systems and taxa while increasing realism?

Q4. In what circumstances could species' traits be leveraged to inform connectivity models? And does this approach improve connectivity conservation?

Q5. Under what circumstances should anisotropy be explicitly incorporated into connectivity models?

N1. Evaluate the ability of methods to capture a range of species' connectivity needs and ecological processes.

N2. Evaluate the importance of key uncertainties such as resistance values.

N3. Compare multi‐species methods in terms of their efficiency (cost, effort) and effectiveness (biological realism).

N4. Link results from connectivity modeling efforts directly to alternative conservation strategies for maintaining and improving connectivity now and into the future.

N5: Invest in computing tools, with a focus on equitable access to the tools needed to conduct large‐scale, validated connectivity analyses.

N6: Research to link connectivity analyses with decision science, to better achieve overall biodiversity conservation goals.

N7: Protocols and workflows for connectivity analysis, including data and code, need to be open, transparent, and reproducible.

Governments and conservation non‐governmental organizations (NGOs) around the world have acknowledged that on‐the‐ground action is needed sooner rather than later to meet convention on biodiversity framework targets aimed at maintaining and restoring ecological connectivity in priority areas and for vulnerable species (e.g., CBD, 2023; World Wildlife Fund, 2024). Consequently, while we continue to address gaps in our understanding, we must also take timely action using the best available information when necessary. For the time being, in cases where data are missing but decisions are needed, conservation practitioners may have to resort to simpler models that may potentially lack biological realism. For example, in comparisons of single, surrogate, and multiple focal species approaches, surrogates did not capture the connectivity and habitat needs of communities, while generic species did better, and species agnostic models yielded variable results (Krosby et al., 2015; Wood et al., 2022). Precaution is needed when implementing connectivity planning based on models with limited certainty. In these cases, it is especially prudent to take the precautionary principle to prevent the loss of potential key habitats and movement corridors. Some approaches can help mitigate knowledge gaps, such as using naturalness and species‐agnostic connectivity models (Marrec et al., 2020) or applying allometric rules (Hartfelder et al., 2020). There are certainly trade‐offs with increasing realism in connectivity modeling and how much realism is necessary for conservation remains uncertain. Finding the balance between increasing the biological realism of connectivity models for specific species and regions and finding appropriate generalizations across multiple species and ecosystems is an important area of future study (Wood et al., 2022).

In situations with very limited time and resources available for modeling, it may be necessary to accept unquantified uncertainty and adopt simple strategies. Examples include: prioritizing protection and/or restoration of larger habitat patches or those adjacent to existing protected areas (Barnes et al., 2018), riparian corridors (de la Fuente et al., 2018), corridors identified in structural connectivity analyses, or areas important for connectivity identified for single species of concern, umbrella species, or generic species (Hanson et al., 2022; Kool et al., 2013). Although all connectivity models should be validated, in these cases of high uncertainty, validation is crucial to ensure sound models inform conservation planning. Furthermore, decisions about which models and data to use in connectivity analysis are context‐driven. In highly intact, homogenous landscapes, connectivity models may not be essential because organisms would be less limited in options when moving. Conversely, in highly modified landscapes with little in the way of natural areas remaining, simpler structural models may be just as discerning as species‐specific models due to the limited areas available for movement.

Another consideration is the need to improve how we communicate uncertainty in both the available data and the results to decision‐makers. Connectivity analyses involve many choices about data, models, methods, metrics and analytical techniques (Diniz et al., 2020; Hilty et al., 2020; Rudnick et al., 2012; Vasudev et al., 2015; Wood et al., 2022; Zeller et al., 2012). Throughout the process, assumptions are made but uncertainty is often not acknowledged nor accounted for (Diniz et al., 2020; Fahrig et al., 2021; Rudnick et al., 2012; Zeller et al., 2012). Placing connectivity analysis in the overall decision context can also be helpful. Decision‐making in the face of uncertainty can be facilitated by clearly recognizing the fundamental objective (Bower et al., 2018; Gregory et al., 2021; Schwartz et al., 2018). If the fundamental objective is unclear, techniques from structured decision‐making can help with clarification (Gregory et al., 2012). Regardless of the methods chosen, it would be beneficial to have clear and complete descriptions of how connectivity models were produced. This can allow easier comparison of outputs and can make the work more useful for researchers and practitioners alike. In other words, connectivity scientists should strive to increase the transparency, openness, and reproducibility of our science (Riordan‐Short et al., 2023). This will improve the reliability of, and confidence in, our science for direct use in conservation action (Roche et al., 2021). Ensuring these qualities should be the responsibility of researchers themselves rather than end‐users. Thus, we encourage researchers to consult helpful checklists (e.g., Jenkins et al., 2023; Parker et al., 2018; Riordan‐Short et al., 2023) and to follow best practices in scientific computing (McIntire et al., 2022; Wilson et al., 2017), including making data and code openly available (Culina et al., 2020; Jenkins et al., 2023; Roche et al., 2015). The benefits of these practices would be further augmented by efforts that emphasize actionable science models developed through co‐production with stakeholders (see Parrott et al., 2019 for example).

Although it is important for land managers to act now to implement connectivity conservation actions, as discussed above, there will be many situations where the use of more realistic models and multiple methods will still be required not just to halt the decline of biodiversity, but also to reverse it. Even if countries can meet the new Global Biodiversity Framework (CBD 2023) and effectively conserve 30% of their lands and waters, that will leave the vast majority of each country being subjected to anthropogenic disturbances. Research has demonstrated that movement by many species, and therefore their connectivity needs, are significantly affected by anthropogenic disturbances (Tucker et al., 2018). Species at risk often have unique needs and/or higher sensitivity to anthropogenic impacts that may not be captured using simpler models (Di Marco et al., 2018; Fraser et al., 2018; Yesuf et al., 2021). In addition, broader landscape planning for multiple species, climate change, and cumulative impact assessments will undoubtedly still require the use of more realistic connectivity models (Liang et al., 2023; Robertson et al., 2018; Tarabon et al., 2019).

The advances and opportunities we present could help collaboration between researchers and practitioners addressing time‐sensitive conservation issues, while the outstanding questions and research priorities that emerged from this review (summarized in Box 1) could guide researchers, governments, and conservation NGOs. Thanks to a decade of advances, connectivity researchers are well‐positioned to help with conservation decisions needed today, while continuing to advance our understanding and confidence in connectivity science.

## AUTHOR CONTRIBUTIONS

**Amanda R. Liczner:** Conceptualization (equal); methodology (equal); project administration (lead); visualization (lead); writing – original draft (equal); writing – review and editing (equal). **Richard Pither:** Conceptualization (equal); funding acquisition (equal); methodology (equal); project administration (equal); supervision (equal); writing – original draft (equal); writing – review and editing (equal). **Joseph R. Bennett:** Investigation (equal); methodology (equal); project administration (equal); writing – original draft (equal); writing – review and editing (equal). **Jeff Bowman:** Investigation (equal); methodology (equal); project administration (equal); writing – original draft (equal); writing – review and editing (equal). **Kimberly R. Hall:** Investigation (equal); methodology (equal); project administration (equal); writing – original draft (equal); writing – review and editing (equal). **Robert J. Fletcher Jr:** Investigation (equal); methodology (equal); project administration (equal); writing – original draft (equal); writing – review and editing (equal). **Adam T. Ford:** Conceptualization (equal); funding acquisition (equal); investigation (equal); methodology (equal); project administration (equal); supervision (equal); writing – original draft (equal); writing – review and editing (equal). **Julia L. Michalak:** Investigation (equal); methodology (equal); project administration (equal); writing – original draft (equal); writing – review and editing (equal). **Bronwyn Rayfield:** Investigation (equal); methodology (equal); project administration (equal); writing – original draft (equal); writing – review and editing (equal). **Julian Wittische:** Methodology (equal); project administration (equal); writing – original draft (equal); writing – review and editing (equal). **Jason Pither:** Conceptualization (equal); funding acquisition (equal); investigation (equal); methodology (equal); project administration (equal); supervision (equal); writing – original draft (equal); writing – review and editing (equal).

## CONFLICT OF INTEREST STATEMENT

The authors have no conflicts of interest to declare.

## Data Availability

No data were used in the creation of this review.
